# Impact of different types of entrepreneurial alertness on entrepreneurial opportunities identification

**DOI:** 10.3389/fpsyg.2022.888756

**Published:** 2022-12-19

**Authors:** Zhenning Li, Bing Jiang, Shulei Bi, Jing Feng, Qianyi Cui

**Affiliations:** ^1^Business School, Shandong University of Technology, Zibo, China; ^2^Doctoral School of Entrepreneurship and Business, Budapest Business School, Budapest, Hungary; ^3^Higher School of Industrial Policy and Entrepreneurship, Peoples' Friendship University of Russia, Moscow, Russia; ^4^Business School, Chinese University of Hong Kong, Hong Kong, Hong Kong SAR, China

**Keywords:** entrepreneurial alertness, entrepreneurial bricolage, entrepreneurial passion, opportunities identification, new ventures

## Abstract

In the context of resource constraints, how different dimensions of entrepreneurial alertness affect the entrepreneurial opportunity recognition of new ventures is an important issue worth studying. From entrepreneurial cognition theory and bricolage theory perspectives, we systematically investigate the intrinsic relationships among entrepreneurial alertness, entrepreneurial bricolage, entrepreneurial passion, and entrepreneurial opportunity recognition. Further, it explored the intrinsic mechanism of role in exploring entrepreneurial opportunity recognition. This study applied stepwise regression analyses and the Bootstrap method to test the hypotheses on a sample of 295 questionnaires of the new venture. The findings revealed that entrepreneurial alertness is positively related to entrepreneurial opportunity recognition. Entrepreneurial bricolage is positively related to entrepreneurial opportunity recognition. Entrepreneurial bricolage partially mediates between entrepreneurial alertness and entrepreneurial opportunity recognition. Entrepreneurial passion positively moderates the relationship between entrepreneurial bricolage and entrepreneurial opportunity identification. The study guides new ventures to enhance entrepreneurial alertness and reasonably use entrepreneurial bricolage to explore entrepreneurial opportunities.

## Introduction

In recent years, the advancement and application of the Internet and other emerging technologies have induced a new wave of entrepreneurship worldwide (Rippa and Secundo, 2019; Sahut et al., 2021). At the same time, China has also put forward a series of policies to encourage entrepreneurship (He et al., 2019). With the support of national policies, the number of new ventures in China has reached record highs, playing a heavy role as a leader and participant in the venture wave. According to the Global Entrepreneurial Ecosystem Index 2020 report released by Startup Blink, a global entrepreneurship research firm, China ranks 14th out of 100 for entrepreneurship globally, ranking first in Asia. According to the latest global survey published on the USNEWS website, China ranks 3rd among the “World's Best Countries to Start a Business in 2020”. These international studies show that the overall entrepreneurial ecosystem in China has been relatively good in recent years. However, the Global Entrepreneurship Monitor (GEM) China Report 2018/2019 points out that its venture failure rate is also rising despite the increasing innovation and internationalization of China's entrepreneurial activities. Especially after the impact of the epidemic and the global economic downturn, the survival environment for new ventures is even less optimistic (Bacq and Lumpkin, 2021). According to the 2020 China Enterprise Development Data Annual Report, there were 1,042,800 enterprise cancellations and revocations in China in 2020, 18.6% year-on-year, the highest number in recent years. On the one hand, due to the increased risk and uncertainty of entrepreneurial activities and the “new entrant defect” of new ventures (Lin et al., 2019; Roy, 2020), and on the other hand, the rapid development of economic transformation, knowledge economy, and big data has intensified the change of China's entrepreneurial environment from relatively stable and orderly to highly dynamic and complex (Bennett and Lemoine, 2014). This situation also makes the survival and development of new ventures more difficult. Under such circumstances, it is a real issue to be discussed in the industry to study how entrepreneurial teams can succeed in the complex and changing external environment and their weaknesses.

Entrepreneurship is a dynamic and changing process, full of opportunities and challenges, and effective identification of entrepreneurial opportunities is one of the essential competencies for successful entrepreneurs (Busenitz et al., 2014; Rocha et al., 2015; Shepherd et al., 2017; Maran et al., 2021). However, opportunity identification is a complex cognitive process influenced by numerous factors (Morris and Liguori, 2016). According to the entrepreneurial cognitive school of thought, entrepreneurial alertness affects an individual's cognitive framework (Lanivich et al., 2022). According to (Foss and Klein, 2010), entrepreneurial alertness helps firms identify market gaps and discover entrepreneurial opportunities in the environment. Tang et al. (2012) also suggest that entrepreneurial alertness makes clever connections between the firm's fragmented old and new knowledge. This correlation further facilitates the generation of new ideas and opportunities. From a mental process perspective, entrepreneurial alertness can be deconstructed as a mental work task beginning with scanning and searching moving through association and connection to evaluation and judgment. Existing research widely recognizes the critical role of entrepreneurial alertness in the opportunities identification process (Masango and Lassalle, 2020), and studies on the relationship between the two based on Chinese entrepreneurial scenarios do exist. However, most studies develop entrepreneurial alertness as a single holistic concept. They lack empirical analysis of the impact of different cognitive dimensions of entrepreneurial alertness on entrepreneurial opportunity identification. Therefore, exploring the mechanism and path of entrepreneurial alertness on entrepreneurial opportunities identification based on entrepreneurial cognitive processes in the Chinese entrepreneurial context is essential.

The research on the role of entrepreneurial alertness in entrepreneurial opportunity identification mainly focuses on the “opportunities discovery view.” However, it lacks empirical research on the mechanism of the inner role of both from the “opportunities creation view,” which is difficult to answer, and “how opportunities are created”. Resources significantly influence the creation of opportunities, and only the reasonable matching and utilization of resources can effectively develop entrepreneurial opportunities (Timmons et al., 2004). Baker and Nelson (2005) proposed the theory of entrepreneurial bricolage by studying several entrepreneurial ventures. The theory suggests that entrepreneurial bricolage is a practical resource strategy for new ventures facing resource constraints. It helps them explore new entrepreneurial opportunities by matching and reconfiguring limited resources. From the perspective of opportunities resource integration, Witell et al. (2017) found that internal resource bricolage can create entrepreneurial opportunities. It explained the opportunities-creating role of entrepreneurial bricolage. Thus, entrepreneurial bricolage provides a new theoretical perspective for the “opportunities creation view”. In summary, entrepreneurial bricolage plays a vital role as a bridge between entrepreneurial alertness and entrepreneurial opportunities identification in new ventures. In addition, Santos S. C. et al. (2020) argue that psychological and emotional factors have a significant impact on entrepreneurial opportunity identification. And when new experiences identify entrepreneurial opportunities through entrepreneurial bricolage, they need to be highly sensitive to information about the external environment and need solid entrepreneurial passion in the face of innovation pressure and risk. It can provide continuous endogenous motivation and emotional support for entrepreneurs to overcome difficulties (Santos and Cardon, 2019). Entrepreneurial passion is an essential influencing factor in the entrepreneurial process and has been affecting the entrepreneurial process. It can enhance the entrepreneur's ability to withstand difficulties and persevere for a more extended period (Cardon et al., 2009b). At the same time, a high level of passion can also enhance the creative use of resources and integration of the entrepreneurial enterprise. New ventures face more pressure in the entrepreneurial process due to the barriers caused by the “new entrant defect,” so they need the psychological support of entrepreneurial passion. Therefore, entrepreneurial passion can play a moderating role in the relationship between entrepreneurial bricolage and entrepreneurial opportunity identification.

Based on entrepreneurial cognition theory and entrepreneurial bricolage theory, this study constructs a theoretical model of entrepreneurial alertness, entrepreneurial bricolage, and entrepreneurial opportunity identification for new ventures, contributing to theory and practice mainly in the following aspects.

- Helping new ventures solve entrepreneurial opportunity identification and make more targeted suggestions and measures. It also reveals the mechanism of the role of different dimensions of entrepreneurial alertness on entrepreneurial opportunity recognition, which makes up for most of the previous studies on the relationship between the two based on single-dimensional entrepreneurial alertness (Neneh, 2019; Daniel et al., 2021; Ramoglou, 2021). It enriches and improves the theory of entrepreneurial cognition (Mitchell et al., 2007; Sassetti et al., 2018; Gancarczyk and Ujwary-Gil, 2021).
- Based on the resource orchestration theory (Sirmon et al., 2011; Huy and Zott, 2019), we investigate the differential mediation paths between the planned and improvised bricolage models in the relationship between entrepreneurial alertness and entrepreneurial opportunity recognition. The findings unlock the “black box” of the intrinsic mechanism of entrepreneurial alertness driving entrepreneurial opportunity recognition and better answer the question of “how entrepreneurs identify and create entrepreneurial opportunities that are not easily recognized by others” (Ireland, 2003; Companys and McMullen, 2007; Li et al., 2020a).
- The article analyzes the moderating effect of entrepreneurial passion on the relationship between entrepreneurial bricolage and entrepreneurial opportunity recognition. The findings enrich the gaps in prior research on the impact of entrepreneurial passion on the relationship (Collewaert et al., 2016; Boone et al., 2020; de Mol et al., 2020).

## Theoretical basis and research hypotheses

### Entrepreneurial alertness

Entrepreneurial alertness is a specific mental model that drives entrepreneurial teams to sift and process internal and external information to identify entrepreneurial opportunities (Gaglio and Katz, 2001; Valliere, 2013). Entrepreneurial alertness is an essential influencing factor in the entrepreneurial process, which affects the entrepreneur's information processing and perception of the industry, which in turn affects the identification of external opportunities. (Ekelund and Kirzner, 1974; Brown et al., 2019). In recent studies, entrepreneurial alertness is considered a complex cognitive process in which individuals actively identify entrepreneurial opportunities (Montiel-Campos, 2021; Cavaliere et al., 2022; Crespo et al., 2022). Tang et al. (2012) define entrepreneurial alertness as a mental activity from scanning for new information to associating with heterogeneous information from different sources and evaluating potential entrepreneurial opportunities, including scanning and search, association and connection, and evaluation and judgment. This paper will analyze the relationship between the different dimensions of entrepreneurial alertness on entrepreneurial opportunities identification and their differences.

### Entrepreneurial bricolage

Entrepreneurial bricolage is a resource utilization strategy for firms in a resource-constrained dilemma. It advocates the creative use of the limited resources at hand to solve new problems or create new opportunities under resource constraints (Phillips and Tracey, 2007; Hooi et al., 2016; Rahman et al., 2020). It breaks the established direction of the utilization and application mode of the resources the enterprise has at hand (An et al., 2018). Previous studies have different dimensional divisions of entrepreneurial bricolage. Baker and Nelson (2005) found two forms of entrepreneurial bricolage: selective bricolage and parallel bricolage. Selective bricolage refers to a focused scrapping strategy that is implemented selectively and focused on individual projects and some areas. Parallel bricolage is a decentralized strategy of simultaneous and collaborative implementation in multiple projects and areas. In addition, the behavioral nature of entrepreneurial bricolage suggests that it can be improvised or planned (Baker et al., 2003). This study will draw on previous scholarly research on whether a plan is set before bricolage, using planned and improvised bricolage dimensions.

### Entrepreneurial passion

Social psychology considers passion as a strong positive emotion (Barrett, 2013). In entrepreneurship, scholars have divided entrepreneurial passion into individual and team-level meanings. The individual level considers entrepreneurial passion as a positive emotion held by individual entrepreneurs toward entrepreneurship, which can enhance the entrepreneurial identity of individuals and act accordingly (Vallerand et al., 2003). The team level views entrepreneurial passion as a collection of entrepreneurial passions of team entrepreneurs that individual members form through shared emotions and identities (Cardon et al., 2017). It contains the collection and intersection of each member's entrepreneurial passion and the manifestation of members' entrepreneurial passion differences (Cardon et al., 2009a). Current research on entrepreneurial passion is not conclusive about entrepreneurship's positive or adverse effects. However, the vast majority of studies have concluded that entrepreneurial passion positively impacts the entrepreneurial process. For example, entrepreneurship can increase the willingness of entrepreneurs to start a business. This increase can directly affect or be achieved through certain mediating variables such as entrepreneurial self-efficacy (Biraglia and Kadile, 2017; Hou et al., 2019).

### Entrepreneurial opportunities identification

Entrepreneurial opportunity identification is a central issue in the entrepreneurial process (Yu et al., 2020), which first originated in the discussion of factors influencing economic equilibrium in economics. Stevenson and Jarrillo-Mossi (1986) argue that opportunity identification is the ability of entrepreneurs to integrate resources and create value. Ardichvili et al. (2003) argue that opportunity identification is integrating and processing ideas and elements of entrepreneurship into a viable business plan. With the continuous development of research, newer studies generally consider the nature of entrepreneurial opportunity identification as a complex cognitive process that includes multiple processes, such as identifying opportunities, recognizing opportunities (Mueller and Shepherd, 2016), and integrating resources. Various factors influence entrepreneurial opportunity identification (Steffens et al., 2022). Regarding the relationship between entrepreneurial alertness and opportunity recognition, Kirzner (2009)suggested that alert entrepreneurs can better exploit information asymmetries to identify entrepreneurial opportunities better. From the perspective of experience affecting entrepreneurial opportunity recognition, Shane (2003) argue that entrepreneurs' a priori knowledge and cognitive characteristics determine that some of them can identify entrepreneurial opportunities that others do not find. Lumpkin and Lichtenstein (2005) argue that entrepreneurs or entrepreneurial teams that pay attention to organizational learning can improve the likelihood of opportunity recognition.

### Entrepreneurial alertness and entrepreneurial opportunities identification

Scanning and search is the search behavior of new ventures to detect changes in the external environment and discover further entrepreneurial information, a precognitive activity to obtain additional information unknown to others, covering both scope and depth (Sassetti et al., 2022). Urban and Wood (2017) found through their study that vigilant mining and exploration of external knowledge, experience, and resources, among others, can enhance a firm's ability to innovate and further uncover new connotations of the explored resources. Newly discovered information catalyzed by the established knowledge and experience of the new ventures constructs and develops the entrepreneurial team's unique cognitive framework for new entrepreneurial opportunities, forming the logic and route of thinking for entrepreneurial opportunities identification. On the one hand, extensive scanning and searching can help entrepreneurs find new information that others have overlooked, expanding their information channels and storage (Tang, 2016). Thus it will improve entrepreneurs' problem-solving and thinking skills and thus better identify the entrepreneurial opportunities contained in the information. Another hand, a deep scanning search can continuously explore, track, and mine the complex information and knowledge deeply embedded in specific industries and segments (Amato et al., 2017). Deep scanning and search indicate a more in-depth scanning and searching of information in a specific direction, industry, or field, with a certain degree of planning and purpose. It has higher accuracy and effectiveness than aimless scanning search, which can help entrepreneurial teams better identify the entrepreneurial opportunities behind complex information.

Association and connection are the cognitive activity of linking two or more previously unrelated pieces of information and further analyzing the correlation between this seemingly unrelated information (Chavoushi et al., 2021), which explains how to apply or extend the collected information. In entrepreneurship, it is difficult for entrepreneurs to identify valuable opportunities from a single piece of information, and how to associate and utilize various information becomes the key to entrepreneurial opportunity identification (Baron and Ensley, 2006). In this case, entrepreneurs must break through the fixed cognitive way and conceptualize and diversify the information based on their cognitive structure of thinking patterns. Through association and connection, entrepreneurs can break the boundaries of thinking and correlate seemingly unrelated things and information (Lee et al., 2016), which is likely to create new valuable information and help companies explore new entrepreneurial opportunities.

Evaluation and judgment refer to the cognitive activity of new ventures to use their existing knowledge base and entrepreneurial cognition to deeply examine whether they have potential business opportunities by analyzing the possible application value of further information (Kadile and Biraglia, 2022). In the entrepreneurial process, as entrepreneurs need to face objective and complex information, purposeless and unsupported selection and utilization of information and resources will waste too much time and energy (de Carolis and Saparito, 2006; Narayanan et al., 2021), failing to identify potential opportunities or missed opportunities due to “windows of opportunity”. Evaluation and judgment are like a filter that helps entrepreneurs filter out more useless information and keep their energy focused on valuable information related to market trends (Pirhadi et al., 2021), which helps entrepreneurs accurately identify potential opportunities (Tang et al., 2012). Therefore, the richer the heterogeneous information scanned and searched, the more frequently the entrepreneurial team evaluates and judges the new information. The more profound the entrepreneurial cognitive stimulation received, the more valuable the filtered information is, and the more accurate the entrepreneurial opportunities identification is (Ardichvili et al., 2003). Therefore, the assessment and judgment facilitate entrepreneurial opportunities identification through post-cognitive information processing. Thus, we propose that.

H1: Entrepreneurial alertness has a positive effect on entrepreneurial opportunity identification.
H1a: Scanning and searching positively affect entrepreneurial opportunities identification.
H1b: Association and connection positively affect entrepreneurial opportunities identification.
H1c: Evaluation and judgment positively affect entrepreneurial opportunities identification.

### Entrepreneurial alertness and entrepreneurial bricolage

Scanning and search provide an immediate basis for identifying and selecting the direction of entrepreneurial bricolage by obtaining information and knowledge about the external environment, such as the market and technology, on a large scale and at a deep level. First, scanning and searching can search diverse and heterogeneous information resources from different industries and fields through multiple channels such as business, social, and innovation networks. This broadens the experience width and business horizon of entrepreneurial teams (Li, 2013), helps to explore potential entrepreneurial opportunities in multiple industries and fields simultaneously, and enriches entrepreneurial teams' entrepreneurial cognition of different entrepreneurial opportunities. So it accelerates the entrepreneurial and cognitive construction of ideas, models, and paths for new ventures (Fatoki, 2014) and points out the direction of entrepreneurial bricolage for creative integration and utilizing existing resources. Therefore, large-scale scanning and search provide more directional options for improvised and planned bricolage and promote the entrepreneurial assemblage activities of new ventures. Secondly, deep scanning and search rely more on non-public information channels (van de Sandt and Mauer, 2019). It can deeply access high-value information resources that others overlook through continuous attention and tracking of specific industries and fields (Campos, 2021), a cognitive learning process for entrepreneurial teams to deeply explore specific potential entrepreneurial opportunities. This process facilitates the precise association and matching of limited resources to entrepreneurial opportunities. It promotes new ventures targeting resources to specific entrepreneurial opportunities (Lumpkin and Lichtenstein, 2005). As a result, deep scanning and search with targeting are more conducive to obtaining critical and scarce information and knowledge required for opportunities development. It provides more precise directions for improvised and planned bricolage. Thus, deep scanning and search stimulate improvised and planned bricolage activities. Thus, deep scanning and search stimulate improvised and planned bricolage activities.

Association and connection are entrepreneurial cognitive behaviors that connect, combine and reconfigure previously unrelated information and knowledge for new ventures (Mole et al., 2019). Its essence is creativity and imagination oriented toward potential entrepreneurial opportunities and how they use their resources. It helps entrepreneurial teams to break out of their inherent cognitive constraints and look at the value attributes and practical uses of existing resources in an alternative way (Salunke et al., 2013), which can effectively stimulate the entrepreneurial bricolage activities of new ventures. In the above process, the entrepreneurial team, on the one hand, realizes the dynamic docking and matching of the limited resources of the enterprise with different application fields and scenarios through the cross-border association of information and knowledge of multiple industries or fields and forms a diversified combination of innovative resources utilization (Mole et al., 2019). On the other hand, focused association and connection of information and learning from the same industry or field promotes the conception and design of the use of limited resources in specific application fields and scenarios. It can identify possible new business models in the future by discovering and establishing new connections, new structures, and new uses among existing resources (Pidduck et al., 2020), meeting the resource requirements for the development of specific potential entrepreneurial opportunities. As a result, the highly alert association and connection inspired improvised and planned bricolage in new ventures through cross-border learning and focused learning.

When new ventures face a multitude of further information and knowledge from multiple industries or fields, the ability to assess their intrinsic application value in a timely and correct manner is critical for them to be the first to take precise entrepreneurial action (Smith et al., 2009; Olugbola, 2017; Kirtley and O'Mahony, 2020). Evaluation and judgment act as a filter for new information and knowledge in the external environment for comparative analysis and argumentative evaluation (Uygur, 2019). It can help entrepreneurial teams sift through all kinds of new information and knowledge to effectively discover the direction and way to use existing resources and promote new entrepreneurial bricolage activities of new ventures. On the one hand, because new ventures have limited resources, too much of a bricolage direction will disperse the resources invested in the business and increase the risk of business failure (Sunduramurthy et al., 2016). Therefore, the evaluation and judgment will consider the firm's resources, analyze its feasibility, and then choose the entrepreneurial collocation direction with a higher success rate. On the other hand, new venture entrepreneurship is a dynamic learning process of gradually exploring the needs of an unknown industry or field (El-Awad et al., 2017; Cosenz and Noto, 2018). Therefore, the evaluation and judgment drive the startup team to continuously optimize and adjust the previous bricolage solution by acquiring new information and knowledge. Such adjustments enable new ventures to be more precise about the direction of the bricolage and improve the efficiency and effectiveness of using available resources. Therefore, we hypothesize:

H2: Entrepreneurial alertness positively affects the entrepreneurial bricolage of new ventures.
H2a: Scanning and search positively affect improvised and planned bricolage.
H2b: Association and connection positively affect improvised and planned bricolage.
H2c: Evaluation and judgment positively affect improvised and planned bricolage.

### Entrepreneurial bricolage and entrepreneurial opportunities identification

Improvised bricolage is motivated by problem orientation, focusing on and responding to “immediate problems” (Wang et al., 2021). In multiple constraints, improvised bricolage can comprehensively integrate material, human, technological, market, and institutional resources, allowing companies to gain more extensive opportunities (Baker, 2007). At the same time, they can creatively reorganize and “smartly” allocate resources in multiple fields to solve problems and breakthrough difficulties (Onwuegbuzie and Mafimisebi, 2021). This situation requires a well-developed knowledge framework and a high degree of cognitive flexibility from those performing the improvised bricolage to actively identify and explore idle resources' potential value and opportunities (Welter et al., 2016). Second, since improvised bricolage is effectual reasoning, improvisational decision logic dominates (Yang, 2018). Open and flexible improvisation under limited rationality is often accompanied by innovation which can produce unpredictable creative results and generate new markets and services in resource reconstruction of entrepreneurial activities (Shabbir et al., 2021), facilitating the discovery and creation of new opportunities for new ventures. Finally, improvised bricolage allows for the simultaneous development of multiple project bricolage activities, strengthening the improvised bricolage's intentional or unintentional collection of information about “potentially useful” resources in different fields (Mair and Marti, 2007), providing a prerequisite for entrepreneurs to identify entrepreneurial opportunities. Thus, improvised bricolage facilitates the identification of entrepreneurial opportunities for new ventures.

Planned bricolage is motivated by opportunity orientation and tends to screen the value of projects before investing in resources (Baker and Nelson, 2005). In assessing the value of a project, planned bricolage rearranges the tacit knowledge in the knowledge architecture (Baron and Ensley, 2006). It breaks the inherent thinking patterns and behavioral inertia to reshape a unique cognitive framework to improve new ventures' opportunity recognition ability and thus increases the probability of identifying opportunities. Second, planned bricolage is based on causal logic. Entrepreneurs enhance their self-learning capabilities and increase their experience-based knowledge through “learning by doing” and trial-and-error learning (Duymedjian and Rüling, 2010). This type of learning deepens the entrepreneur's understanding of the composition of relationships between resources, reshapes subjective knowledge about entrepreneurship, and helps the entrepreneur identify new opportunities. Finally, in terms of effectiveness and cost, the energy and resources of new ventures are limited. Planned bricolage enables real-time adjustment of bricolage solutions in conjunction with the current resources state (Shaheen et al., 2022). It further helps the entrepreneur to choose the most suitable option. This adjustment reduces the costly undertakings caused by aimless bricolage and helps companies save energy to explore new entrepreneurial opportunities (Fisher, 2012). On the basis of the logic and evidence above, we propose that:

H3: Entrepreneurial bricolage has a positive effect on entrepreneurial opportunities identification.
H3a: Planned bricolage has a positive effect on entrepreneurial opportunities identification.
H3b: Improvised bricolage has a positive effect on entrepreneurial opportunities identification.

### The mediating role of entrepreneurial bricolage

In constructing a model of the opportunities recognition process, Ardichvili et al. (2003) suggest that alertness entrepreneurs do not rely solely on a keen perception of the outside world to identify entrepreneurial opportunities. The rational allocation and utilization of resources by the entrepreneur have an essential impact on the success of identifying entrepreneurial opportunities. Thus, resource-constrained creative bricolage plays a crucial role in the process of entrepreneurial alertness influencing opportunity identification. It is because an alert entrepreneur's search for and acquisition of valuable external resources provides the required resource pool for entrepreneurial bricolage (Obschonka et al., 2017; Sharma, 2019). As an entrepreneurial behavior, entrepreneurial bricolage can significantly impact the transformation of entrepreneurial factors into entrepreneurial outcomes for new ventures (Hou et al., 2022). Through the entrepreneurial bricolage, the entrepreneurial firm can analyze and reuse previously valueless human, material, and other resources within the firm (Tindiwensi et al., 2021), thus helping entrepreneurs to discover the new value of resources. It facilitates the entrepreneur to identify or create opportunities. As a result, driven by the perception of entrepreneurial alertness, new ventures can quickly respond to fleeting market opportunities by focusing on entrepreneurial bricolage with the immediate utilization of limited resources (Renko et al., 2012) and can identify entrepreneurial opportunities more efficiently. Thus, entrepreneurial pastiche plays a key mediating role between entrepreneurial alertness and entrepreneurial opportunity identification. Thus, we hypothesize:

H4: The entrepreneurial bricolage mediates between entrepreneurial alertness and entrepreneurial opportunities identification.
H4a: Planned bricolage mediates between entrepreneurial alertness and entrepreneurial opportunities identification.
H4b: Improvised bricolage mediates between entrepreneurial alertness and entrepreneurial opportunities identification.

### The moderating effect of entrepreneurial passion

Successful entrepreneurs often attach importance to the power of entrepreneurial passion (Li et al., 2020b), which represents a strong sense of identity of entrepreneurs. The process of entrepreneurship is full of uncertainty, and the process of piecing together is full of risk and the possibility of failure (Nor-Aishah et al., 2020). Entrepreneurial passion is a positive emotion when entrepreneurs face difficulties (Neneh, 2022). Entrepreneurs with higher entrepreneurial passion will have a stronger inclination toward their goals and are willing to send more significant efforts to achieve their entrepreneurial goals (Syed et al., 2020). When new ventures face resource constraints, entrepreneurial passion becomes a driving force for entrepreneurs to persist in their bricolage activities (Lee and Herrmann, 2021), thereby increasing the frequency and probability of bricolage activities, sustaining the discovery of new value in the resources at hand (Luu and Nguyen, 2021), and increasing the likelihood of opportunity identification. In addition, emotional contagion theory suggests that entrepreneurial passion enhances the entrepreneur's own entrepreneurial identity and gains emotional resonance from other entrepreneurs and investors through emotional contagion (Cardon, 2008; Cardon and Kirk, 2015). It can help companies broaden their social networks and access social capital, helping them conduct bricolage activities more efficiently and facilitating entrepreneurial opportunity identification for new ventures. These arguments lead us to hypothesize that:

H5: Entrepreneurial passion plays a positive moderating role between entrepreneurial bricolage and entrepreneurial opportunities identification.
H5a: Entrepreneurial passion positively moderates the role between planned bricolage and entrepreneurial opportunities identification.
H5b: Entrepreneurial passion positively moderates the role between improvised bricolage and entrepreneurial opportunities identification.

**Figure 1:**
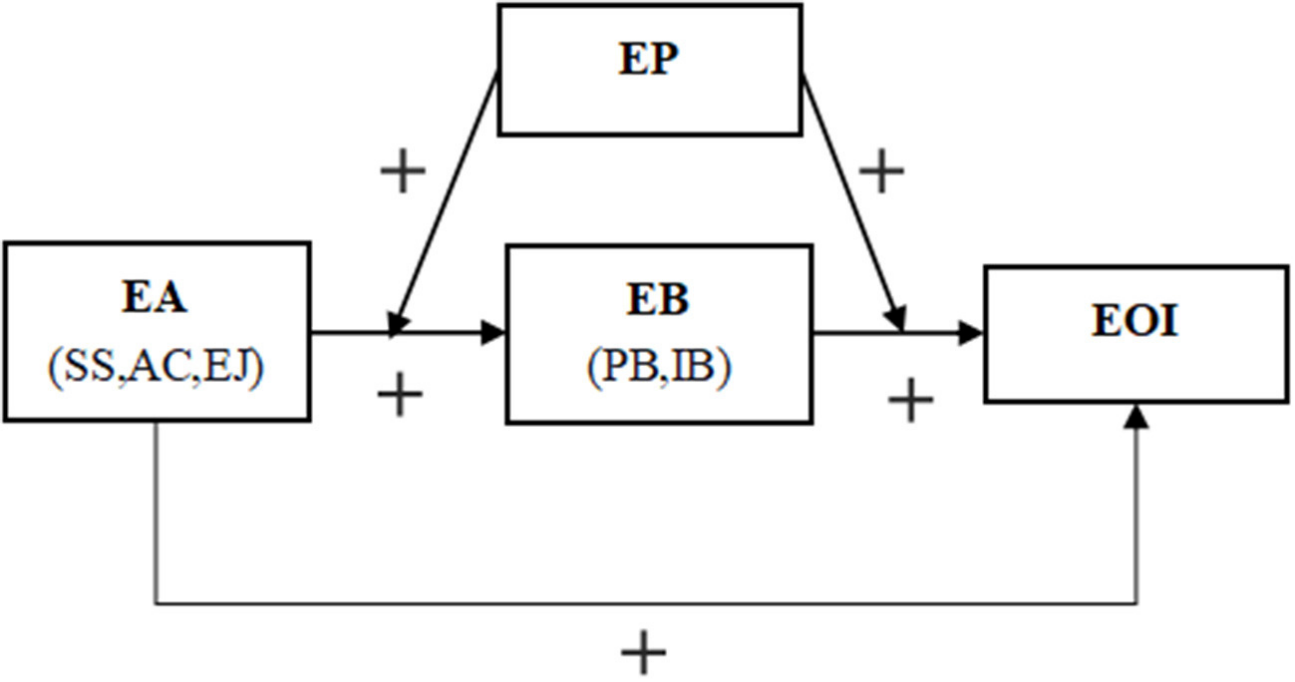
Theoretical model.

The previous hypothesis shows that entrepreneurial alertness in new ventures acts on entrepreneurial opportunity identification through entrepreneurial bricolage. As a result, we can further deduce that the intensity of the mediating effect of entrepreneurial bricolage varies depending on the level of entrepreneurial passion, i.e., a mediating effect is moderated. It is mainly because entrepreneurial passion, a joint, solid, and positive emotional expression within the entrepreneurial team (Anjum et al., 2021), has a mutual, self-reinforcing, and sustained motivational effect and is a crucial driver of entrepreneurial activity (Feng and Chen, 2020). Therefore, the higher the entrepreneurial passion for new ventures, the more it can give full play to the external environment and the cognitive role of entrepreneurial alertness to guide and stimulate the creative bricolage of the limited resources available and positively promote entrepreneurial opportunity identification. On the contrary, when new ventures lack entrepreneurial passion, their entrepreneurial alertness to the external environment is severely reduced (Bignetti et al., 2021). The lack of environmental information prevents new ventures from effectively planning and implementing entrepreneurial bricolage activities (Guo et al., 2016), inhibiting entrepreneurial opportunity identification. As a result, the higher the entrepreneurial passion, the stronger the positive effect of entrepreneurial bricolage on entrepreneurial opportunity recognition. Thus, the stronger the impact of entrepreneurial alertness on entrepreneurial opportunity recognition transmitted through the mediating effect of entrepreneurial bricolage. Therefore, we hypothesize:

H6: Entrepreneurial passion positively moderates the mediating role of entrepreneurial bricolage in entrepreneurial alertness and entrepreneurial opportunities identification.
H6a: Entrepreneurial passion positively moderates the mediating role of planned bricolage in entrepreneurial alertness and entrepreneurial opportunities identification.
H6b: Entrepreneurial passion positively moderates the mediating role of improvised bricolage in entrepreneurial alertness and entrepreneurial opportunities identification. Our Theoretical model issummarized below in Figure 1.

## Research design

### Data collection and sample

This study uses a questionnaire to collect data and draw on the research results of Zaichkowsky (1985) and Li and Atuahene-Gima (2001). We selected new ventures established for < 8 years as the subjects of the study and distributed questionnaires to executives of these companies who were more knowledgeable about the company. Moreover, we limited the number of respondents to three per company. Regarding the selection of research regions, this study refers to the “2020 China Regional Innovation and Entrepreneurship Index” published by the National Development Institute of Peking University and selects regions with high, medium, and low innovation and entrepreneurship activity for measurement, respectively. The sample covers the provinces and cities of Shandong, Jiangsu, Beijing, and Jilin to reduce the variability caused by the different research regions. The research sample includes several industries such as manufacturing, computer services, commerce and logistics, biopharmaceuticals and catering, education, and leisure and tourism, which better cover new ventures in the region. At the same time, the researchers used a random sampling method to collect data in cooperation with local government agencies and professional market research companies. To ensure the randomness and representativeness of the sample selection process, we randomly selected sample companies from the list of new ventures they provided as research subjects. To ensure the quality of research data, we read much literature in the questionnaire design stage and sort out mature scales of relevant variables. At the same time, the researchers worked with experts and professors in innovation and entrepreneurship research to design the questionnaire, translate the mature English scales item by item, and correct the wording and logic. We selected 10 new venture entrepreneurs or core team members for pre-research in the pre-research phase. The questions were modified according to the feedback from the entrepreneurs to make them more suitable for the Chinese context and national conditions.

In the formal research stage, the research mainly used a combination of face-to-face and email research. Entrepreneurs were contacted according to the contact information on the list of new ventures provided by government agencies and professional research institutions, and we conducted research on companies willing to cooperate with the research. We distributed electronic questionnaires to companies that were not convenient for field research by asking for email addresses. The completed electronic questionnaires are displayed on the website's backend with the completed questionnaires' results. In this study, to avoid the questionnaire being filled out by the same person more than once (online and offline at the same time), we did not issue electronic questionnaires to companies where we conducted on-site interviews. We distributed 600 questionnaires and finally collected 413 questionnaires, a recovery rate of 68.83%. Among the returned questionnaires, we excluded 118 invalid questionnaires that were incomplete or filled in randomly and obtained 295 valid questionnaires, giving a reasonable recovery rate of 49.17%. In addition, we used *t*-tests to compare the differences between participating and non-responding firms in terms of firm size, firm age, and industry type. The results showed no significant differences between the two parts of the questionnaire, indicating that the non-response bias was within a manageable range. Table 1 shows the distribution of sample characteristics.

**Table 1:** Distribution of sample characteristics.

**Variables**	**Category**	**Frequency**	**Frequency (%)**	**Variables**	**Category**	**Frequency**	**Frequency (%)**
Education Level	College and below	58	19.7	Company Age	< 3 years	168	56.9
	Undergraduate	161	54.6		3–5 years	84	28.5
	Master or PhD	76	25.8		6–8 years	43	14.6
Company Size	< 50 people	135	45.8	Industry type	Production manufacturing	65	22.0
	51–100 people	84	28.5		Computer services	77	26.1
	101–300 people	53	18.0		Commercial logistics industry	62	21.0
	301–500 people	18	6.1		Biopharmaceutical industry	25	8.5
	>500 people	5	1.7		Other Industries	66	22.4

### Variable measurement

This study uses the mature questionnaire developed by domestic and foreign scholars for reference and the Likert 7-point scoring method for measurement.

- Entrepreneurial alertness. This study used the scale developed by Tang et al. (2012). Entrepreneurial alertness includes three dimensions: scanning and search, association and connection, and evaluation and judgment, of which the scanning and search dimension has six items, such as “I have frequent interactions with others to acquire new information”, and “I am an avid information seeker. “ The association and connection dimension has three items, which include “I see links between seemingly unrelated pieces of information”, and “I often see connections between previously unconnected domains of information.” Lastly, the evaluation and judgment dimension has four items, two examples of which are “I have a gut feeling for potential opportunities” and “When facing multiple opportunities, I am able to select the good ones”.
- Entrepreneurial bricolage. In this study, we refer to Senyard et al. (2009)'s study and clearly distinguish between the two dimensions of planned and improvised bricolage, with four questions for planned bricolage, such as “I can integrate available resources to create a detailed plan before acting” and “I can act strictly according to the program”. The improvised bricolage dimension has four items, two being “I can successfully address new challenges by integrating resources not intended for this program” and “I can generate novel ideas in action”.
- Entrepreneurial passion. This study draws on the scale (Cardon, 2008) with seven question items. Some of those questions are “I have a passion for creating and improving business development teams” and “I am good at seizing opportunities and enjoy exploring new and uncharted territories”.
- Entrepreneurial opportunity identification. This study draws on An et al. (2018) to measure entrepreneurial opportunity recognition through a 3-item scale. Examples are “I have a special alertness or sensitivity to new opportunities represented by new products and new markets” and “It's easier for us to see potential new opportunities”.
- Control variables. This study draws on previous scholars' research to prevent other factors from interfering with the research results. This paper controls variables such as firm age, size, and industry type. Because entrepreneurs' human capital significantly impacts entrepreneurship and opportunity identification (Ucbasaran et al., 2009), we also control for the education level of entrepreneurs.

## Empirical tests and analysis of results

### Reliability and validity tests

The valid questionnaires were analyzed using SPSS 23.0 and AMOS 24.0 software. Table 2 demonstrates the results of the reliability and validity tests for each variable. The Cronbach's α coefficients of all variables in this study were >0.7. The combined reliability (CR) values were >0.8, indicating the scale has good reliability.

**Table 2:** Reliability and validity tests.

**Variables**	**Title item**	**Loadings**	**Reliability and validity indicators**
Scanning and search	SS1	0.750	α = 0.876; CR = 0.877; AVE = 0.544
	SS2	0.775	
	SS3	0.720	
	SS4	0.737	
	SS5	0.718	
	SS6	0.724	
Association and connection	AC1	0.805	α = 0.867; CR = 0.868; AVE = 0.686
	AC2	0.851	
	AC3	0.828	
Evaluation and judgment	EJ1	0.735	α = 0.839; CR = 0.842; AVE = 0.572
	EJ2	0.764	
	EJ3	0.790	
	EJ4	0.734	
Planned bricolage	PB1	0.759	α = 0.876; CR = 0.876; AVE = 0.639
	PB2	0.836	
	PB3	0.818	
	PB4	0.782	
Improvised bricolage	IB1	0.811	α = 0.860; CR = 0.860; AVE = 0.605
	IB2	0.785	
	IB3	0.758	
	IB4	0.756	
Entrepreneurship passion	EP1	0.710	α = 0.886; CR = 0.887; AVE = 0.529
	EP2	0.722	
	EP3	0.738	
	EP4	0.727	
	EP5	0.744	
	EP6	0.714	
	EP7	0.736	
Entrepreneurial opportunities identification	EOI1	0.742	α = 0.795; CR = 0.795; AVE = 0.564
	EOI2	0.715	
	EOI3	0.793	

Meanwhile, the factor loadings of the measures were all above 0.7, and the AVEs were all >0.5, indicating that the indicators had high convergent validity. According to the data in Tables 2, 3, we can see that the square root of AVE is greater than the correlation coefficient between the variables, indicating that the scale has high discriminant validity. The questionnaires were pre-researched and revised before distribution and mainly used existing mature scales to ensure better content validity. The results of the validation factor analysis showed that χ2 = 759.394, df = 416, χ2/df = 1.825, *p* < 0.001; IFI = 0.928, TLI = 0.918, CFI = 0.927, all >0.900; RMSEA=0.053, < 0.080. It shows that the overall fit of the theoretical model is good.

**Table 3:** Mean, standard deviation, correlation coefficient, and AVE square root values of each variable.

**Variables**	**1**	**2**	**3**	**4**	**5**	**6**	**7**	**8**	**9**	**10**	**11**
1 EL	1.000										
2 CY	−0.016	1.000									
3 CS	−0.035	0.260^**^	1.000								
4 IT	−0.052	0.175^**^	0.055	1.000							
5 SS	0.092	−0.039	0.055	−0.003	**0.738**						
6 AC	0.024	−0.053	0.014	−0.049	0.306^**^	**0.828**					
7 AJ	−0.041	−0.107	0.012	−0.059	0.347^**^	0.275^**^	**0.756**				
8 PB	0.173^**^	−0.089	−0.040	−0.112	0.403^**^	0.403^**^	0.367^**^	**0.799**			
9 IB	0.137^*^	−0.028	0.001	0.002	0.404^**^	0.338^**^	0.402^**^	0.456^**^	**0.778**		
10 EP	0.050	−0.015	0.058	−0.075	0.235^**^	0.178^**^	0.227^**^	0.334^**^	0.351^**^	**0.727**	
11 EOI	0.097	−0.067	−0.011	−0.001	0.402^**^	0.368^**^	0.419^**^	0.476^**^	0.456^**^	0.143^*^	**0.751**
M	2.060	1.580	1.890	2.830	4.781	5.064	4.964	5.003	5.068	5.139	5.092
SD	0.672	0.733	1.013	1.449	0.775	0.875	0.814	0.795	0.834	0.698	0.798

EL, education level; CY, company years; CS, company size; IT, industry type; SS, scanning and search; AC, assocaition and connection; AJ, assessment and judgment; PB, planned bricolage; IB, improvised bricolage; EP, entrepreneurial passion; EOI, entrepreneurial opportunities identification; the same below.

Values in bold font on the diagonal are the square root of AVE.

*n* = 295,

* *p* < 0.05,

** *p* < 0.01.

### Correlation analysis of variables

Table 3 demonstrates the variables' descriptive statistics, correlation coefficients, and differential validity. The results show significant positive correlations between the three dimensions of entrepreneurial alertness and the three variables of planned bricolage, improvised bricolage, and entrepreneurial opportunity identification. There was also a significant positive correlation between planned and improvised bricolage and entrepreneurial opportunity recognition. The research hypotheses were initially verified and provided support for further hypothesis testing.

### Common method deviation and covariance test

Since the same subjects filled in all the variables in this paper, we informed the subjects before filling in the questionnaire that the questionnaire was for academic research only and was filled in two stages. However, this paper is not a longitudinal study in the strict sense, and there is a problem of common method bias. This study controlled for common method bias in three ways, procedurally and statistically. (1) Procedurally, this study used multiple question items to measure different constructs and placed the independent, dependent, and moderating variables in different positions on the questionnaire. (2) Statistically, this study draws on Podsakoff et al. (2003) and other scholars to adopt different statistical instruments to measure common method bias. First, this study used Harman's one-way test. The unrotated exploratory factor analysis results extracted seven factors with characteristic roots greater than one. The variance explained by the largest factor was 29.638%, which was below the 40% criterion and did not show a single factor explaining multiple variances. Therefore, we determined that there was no significant common method bias problem in the sample data. Second, we performed a validated factor analysis by loading all entries on a single factor, referring to the method of Korsgaard and Roberson (1995) to test for common method bias. The results revealed that the one-factor model fit (χ2/df = 6.798, IFI = 0.468, TLI = 0.427, CFI = 0.464, RMSEA = 0.140) was significantly worse than the fit of the 7-factor measurement model used in this study (χ2/df = 1.825, IFI = 0.928, TLI = 0.917, CFI = 0.927, RMSEA = 0.053). We further confirmed that this study had no serious common method bias. Third, this paper adopts the “control unmeasured single method latent factor method”, in which all the entries are loaded onto the original variables. At the same time, these entries are also loaded onto a common variable to compare whether the model fit after controlling for the common method factor is better than the original model. Comparing the leading fit indices of the two models' yields: Δχ2/df = 0.084, ΔSRMR = 0.002, ΔRMSEA = 0.003, ΔCFI = 0.008, and ΔTLI = 0.010, compared to the fit indices of the seven-factor model, we can find that the difference is < 0.02 for both CFI and TLI, and < 0.01 for both SRMR and RMSEA. Therefore, we can judge that the model controlling for common method bias is not significantly better than the original model. In summary, we can judge that there is no serious common method bias among the variables in this investigation. Also, the study conducted multicollinearity tests for all models to avoid the effect of multicollinearity on the test results. The results found that the VIF values of all variables were below three, and the tolerance was >0.1, indicating no severe multicollinearity problem. Therefore the study can use hierarchical regression analysis for hypothesis testing.

### Hypothesis testing results

#### Main effects test

The study used hierarchical regression to verify the relationship between entrepreneurial alertness and entrepreneurial opportunity recognition and test the mediating role of entrepreneurial bricolage and the moderating role of entrepreneurial passion. As shown in Table 4, Model 1 included only control variables. Model 2 added entrepreneurial alertness (scanning and search, association and connection, and assessment and judgment) to Model 1. The explanatory power of this model has increased. The regression results showed that scanning and search, association and connection, and assessment and judgment all had a positive effect on entrepreneurial opportunities identification (*r* = 0.230, *p* < 0.001; *r* = 0.219, *p* < 0.001; *r* = 0.284, *p* < 0.001), and hypothesis H1 (H1a, H1b, H1c) were tested.

**Table 4:** Results of hierarchical regression analysis of intermediary effects.

**Variables**	**Entrepreneurial opportunities Identification**					**Planned bricolage**		**Improvised bricolage**	
	**Model 1**	**Model 2**	**Model 3**	**Model 4**	**Model 5**	**Model 6**	**Model 7**	**Model 8**	**Model 9**
EL	0.097	0.083	−0.001	0.045	0.055	0.167^**^	0.148^**^	0.137	0.122^*^
CY	−0.071	−0.015	−0.038	−0.010	−0.020	−0.068	−0.016	−0.032	0.023
CS	0.011	−0.025	0.010	−0.013	−0.020	−0.011	−0.045	0.014	−0.021
IT	0.016	0.035	0.042	0.054	0.028	−0.090	−0.072	0.014	0.032
SS		0.230^***^		0.168^**^	0.174^**^		0.236^***^		0.242^***^
AC		0.219^***^		0.150^**^	0.176^**^		0.265^***^		0.188^**^
EJ		0.284^***^		0.228^***^	0.220^***^		0.213^***^		0.276^***^
PB			0.341^***^	0.261^***^					
IB			0.299^***^		0.230^***^				
EP									
R^2^	0.014	0.301	0.301	0.348	0.339	0.045	0.317	0.020	0.288
AdjustedR^2^	0.001	0.284	0.287	0.330	0.320	0.032	0.300	0.006	0.271
*F*-value	1.039	17.679^***^	20.712^***^	19.074^***^	18.320^***^	3.455	19.020^***^	1.458	16.605^***^

* Correlation is significant at 0.05 level,

** Correlation is significant at 0.01 level,

*** Correlation is significant at 0.001 level.

#### Test of the mediating role of entrepreneurial bricolage

From model 7, the positive effect of entrepreneurial alertness (scanning and search, association and connection, and evaluation and judgment) on planned bricolage was significant (*r* = 0.236, *p* < 0.001; *r* = 0.265, *p* < 0.001; *r* = 0.213, *p* < 0.001); from model 9, the positive effect of entrepreneurial alertness (scanning and search, association and connection, and evaluation and judgment) on improvised bricolage was significant (*r* = 0.242, *p* < 0.001; *r* = 0.188, *p* < 0.01; *r* = 0.276, *p* < 0.001), and hypothesis H2 (H2a, H2b, H2c) was verified. Meanwhile, the inclusion of the mediating variables planned bricolage and improvised bricolage in Model 3 increased R^2^ by 0.287 and improved the model's explanatory power. Moreover, both planned bricolage and improvised bricolage had a significant positive effect on entrepreneurial opportunity identification (*r* = 0.341, *p* < 0.001; *r* = 0.299, *p* < 0.001). The results validated hypothesis H3(H3a, H3b). Next, the regression of entrepreneurial alertness and entrepreneurial bricolage as independent variables on entrepreneurial opportunity identification. Comparing model 2 and model 4, we can see that the positive effect of planned bricolage on entrepreneurial opportunity identification in model 4 is still significant after adding planned bricolage (*r* = 0.261, *p* < 0.001); and the positive effects of scanning and search, association and connection, and evaluation and judgment on entrepreneurial opportunity identification are still significant at this time (*r* = 0.168, *p* < 0.01; *r* = 0.150, *p* < 0.01; *r* = 0.228, *p* < 0.001). However, the coefficients of the effects were all reduced compared to model 2. Finally, compare model 2 with model 5. After adding improvised bricolage, improvised bricolage significantly positively affects entrepreneurial opportunity identification within model 5 (*r* = 0.230, *p* < 0.001). Moreover, there is still a significant positive effect on scanning and search, association and connection, and evaluation and judgment on entrepreneurial opportunity identification (*r* = 0.174, *p* < 0.01; *r* = 0.176, *p* < 0.01; *r* = 0.220, *p* < 0.001), but the coefficients all decreased. Thus both planned and improvised bricolage partially mediated the effect between entrepreneurial alertness and entrepreneurial opportunity identification, so the results validated hypothesis H4 (H4a, H4b).

#### Test of the moderating effect of entrepreneurial passion

**Figure 2:**
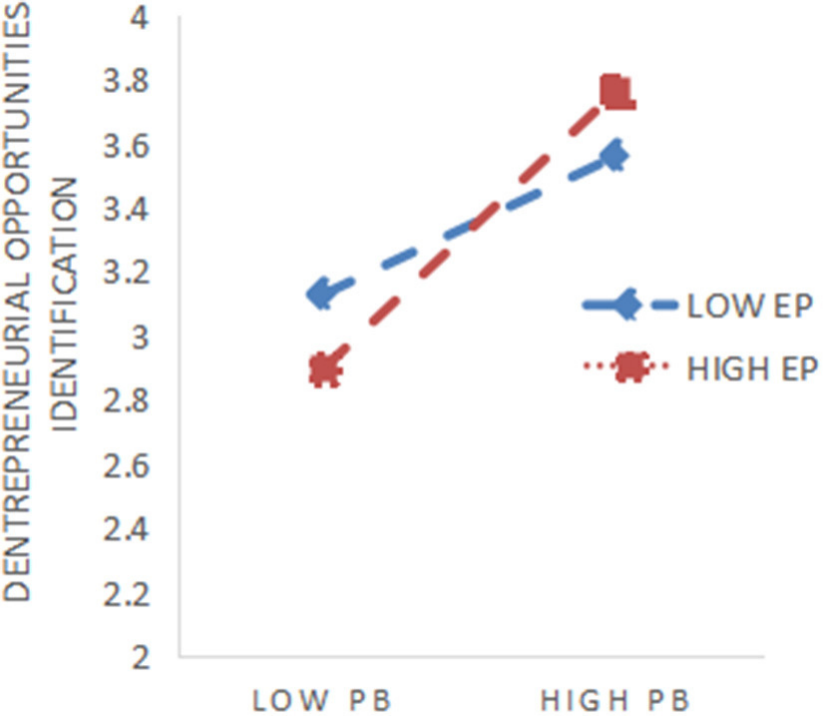
Moderating effect of EP on PB.

**Figure 3:**
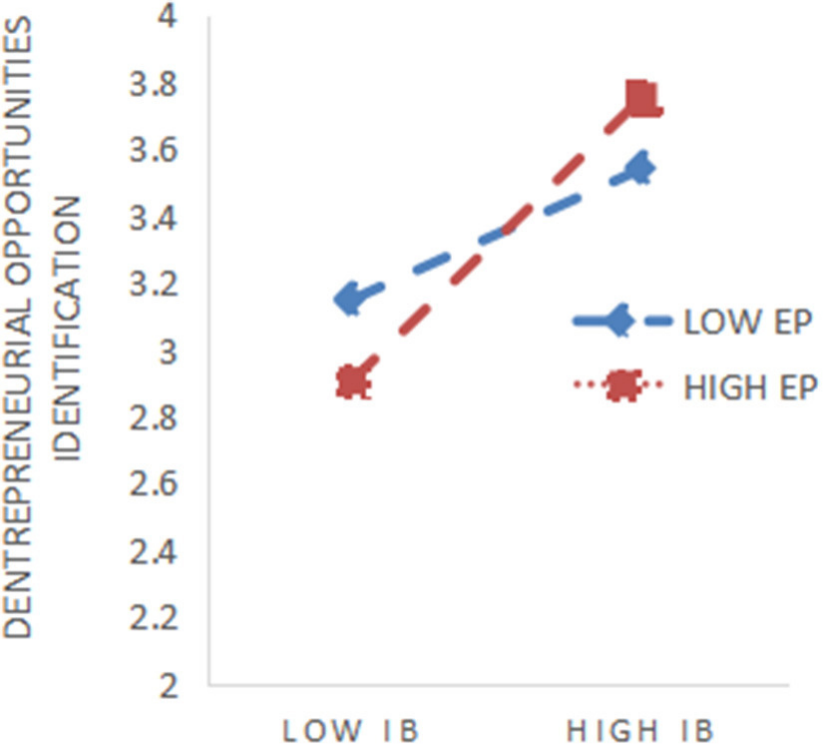
Moderating impact of EP on IB.

The study centralizes the independent and moderating variables to avoid multicollinearity and constructs interaction terms between them. As shown in Table 5, adding the moderating variable entrepreneurial passion to model 1, the results showed that entrepreneurial passion had a negative but insignificant effect on entrepreneurial opportunity identification. Then, adding planned bricolage and entrepreneurial passion in model 2, the results showed that entrepreneurial passion positively moderates the facilitative relationship between planned bricolage and entrepreneurial opportunity identification (*r* = 0.365, *p* < 0.001). Moreover, entrepreneurial passion positively moderates the relationship between improvised bricolage and entrepreneurial opportunity identification in model 4 (*r* = 0.350, *p* < 0.001), so hypothesis H5 (H5a, H5b) was verified. In order to visualize the moderating effect of entrepreneurial passion, the paper plots the moderating effect, as shown in Figures 2, 3.

**Table 5:** Results of hierarchical regression analysis of moderating effects.

**Variables**	**Entrepreneurial opportunities Identification**			
	**Model 1**	**Model 2**	**Model 3**	**Model 4**
EL	0.018	0.050	0.036	0.051
CY	−0.039	−0.045	−0.057	−0.055
CS	0.017	0.017	0.006	−0.000
IT	0.058	0.058	0.008	0.011
SS				
AC				
EJ				
PB	0.482^***^	0.403^***^		
IB			0.456^***^	0.428^***^
EP	−0.015	0.108^*^	−0.019	0.023
PB^*^EP		0.365^***^		
IB*EP				0.350^***^
R^2^	0.231	0.349	0.213	0.333
AdjustedR^2^	0.215	0.333	0.196	0.317
*F*-value	14.451^***^	21.959^***^	12.964^***^	20.457^***^

* Correlation is significant at 0.05 level,

^**^Correlation is significant at 0.01 level,

*** Correlation is significant at 0.001 level.

#### Moderated mediating effects test

In this study, we used PROCESS 3.0 to test the mediating effect with moderation using Bootstrap by adding or subtracting one standard deviation from the mean of the moderating variable entrepreneurial passion for pushing the mediating effect under low and high entrepreneurial passion. Table 6 demonstrates the results of the test. The results show that the indirect effect of each dimension of entrepreneurial alertness on entrepreneurial opportunity identification through planned bricolage is not significant at low entrepreneurial passion (95% confidence intervals of [−0.033, 0.115], [−0.050, 0.091] and [−0.022, 0.108] include 0 respectively). In contrast, at high entrepreneurial passion, the indirect effect of each dimension of entrepreneurial alertness on entrepreneurial opportunities identification through planned bricolage was significant (95% confidence intervals were [0.106, 0.277], [0.094, 0.239], and [0.088, 0.249] respectively, all excluding 0). As entrepreneurial passion increases, the mediating role of planned bricolage between entrepreneurial alertness and entrepreneurial opportunity recognition becomes more pronounced, so H6 a is supported. Under low entrepreneurial passion, the indirect effect of each dimension of entrepreneurial passion on entrepreneurial opportunities recognition through improvised bricolage is not significant (95% confidence intervals are [−0.035, 0.102], [−0.031, 0.075], and [−0.030, 0.095] all including 0, respectively). In contrast, at high entrepreneurial passion, the indirect effect of each dimension of entrepreneurial alertness on entrepreneurial opportunities identification through improvised bricolage was significant (95% confidence intervals were [0.098, 0.276], [0.070, 0.209], and [0.092, 0.254], respectively, all excluding 0). The mediating role of improvised bricolage between entrepreneurial alertness and entrepreneurial opportunity recognition becomes more significant as entrepreneurial passion increases, so H6 b is supported.

**Table 6:** Results of the mediating effect of being moderated (Bootstrap method) test.

**Action Path**	**Grouping of adjustment variables**	**Indirect effects**	**Boot SE**	**95% confidence interval**	
				**Lower limit**	**Upper limit**
SS → PB → EOI	Low EP	0.043	0.038	−0.033	0.115
	High EP	0.180	0.438	0.106	0.277
SS → IB → EOI	Low EP	0.031	0.035	−0.037	0.102
	High EP	0.180	0.045	0.098	0.276
AC → PB → EOI	Low EP	0.026	0.037	−0.050	0.091
	High EP	0.157	0.038	0.094	0.239
AC → IB → EOI	Low EP	0.020	0.027	−0.031	0.075
	High EP	0.135	0.036	0.070	0.209
AJ → PB → EOI	Low EP	0.044	0.033	−0.022	0.108
	High EP	0.155	0.040	0.088	0.249
AJ → IB → EOI	Low EP	0.030	0.032	−0.030	0.095
	High EP	0.168	0.042	0.092	0.254

## Research conclusions and insights

### Research findings and theoretical contributions

This study integrates entrepreneurial cognition theory and bricolage theory to explore the influence of different dimensions of entrepreneurial alertness on entrepreneurial opportunity recognition under resource constraint dilemma as a research perspective. This paper constructs the first theoretical model of the relationship between the variables of entrepreneurial alertness, entrepreneurial bricolage, entrepreneurial opportunity recognition, and entrepreneurial passion and proposes related research hypotheses. Through the empirical analysis of the survey data, the results show that (1) the three dimensions of scanning and search, association and connection, and assessment and judgment positively affect entrepreneurial opportunity recognition. However, to different degrees, evaluation and judgment are the most vital facilitator, scanning and searching for the second strongest and association and connection the smallest. (2) All three dimensions of entrepreneurial alertness significantly affect planned and improvised bricolage. (3) The two dimensions of entrepreneurial bricolage have a significant positive effect on entrepreneurial opportunity recognition, and both play a mediating role between entrepreneurial alertness and entrepreneurial opportunity recognition. (4) Entrepreneurial passion positively moderates the contribution of planned and improvised bricolage to entrepreneurial opportunity identification. (5) Entrepreneurial passion positively moderates the mediating role of entrepreneurial bricolage in entrepreneurial alertness and entrepreneurial opportunity recognition.

The theoretical contributions of this study are as follows. (1) Based on the cognitive perspective of the entrepreneurial process, this study illustrates the process mechanism of transitioning from different dimensions of entrepreneurial alertness to entrepreneurial opportunity recognition, which enriches the theoretical research related to entrepreneurial alertness. The existing studies have focused on the effect of unidimensional entrepreneurial alertness on opportunity recognition, ignoring the variability of the effect of different dimensions (Bacq et al., 2017; Dheer and Lenartowicz, 2019; Gill et al., 2021). This study analyzes and compares the strengths of scanning and search, association and connection, and evaluation and judgment. This finding explains the differences in the pathways of entrepreneurial alertness catalyzing entrepreneurial opportunity recognition. (2) The paper sorted out the differential attributes of planned and improvised bricolage in the opportunity creation process. Furthermore, this study found new methodological options for entrepreneurial opportunity identification in new ventures based on entrepreneurial bricolage theory. Related studies have focused on the opportunity discovery view of entrepreneurial opportunity identification while ignoring the opportunity creation role of the firm's internal resources. This study analyzes the contribution of entrepreneurial alertness to opportunity identification through entrepreneurial bricolage. It also analyzes the role of planned and improvised bricolage in bridging entrepreneurial alertness and opportunity identification. This finding provides an essential theoretical basis for analyzing the mechanisms underlying the role of entrepreneurial alertness in driving entrepreneurial opportunity recognition. (3) The paper analyzes the contribution of entrepreneurial passion to the relationship between entrepreneurial bricolage and entrepreneurial opportunity recognition from the psychological perspective of entrepreneurs. Furthermore, it also clarifies the critical role played by entrepreneurial passion in the process of entrepreneurial bricolage-mediated behavior enriching the research on the influence of entrepreneurial psychology and affective factors on the entrepreneurial process. Cardon and Kirk (2015) showed that entrepreneurial passion is crucial in self-motivation and emotional support when the entrepreneurial team encounters difficulties. It can provide the entrepreneurial team with the motivation to persevere. It can give endless inspiration for the entrepreneurial team to strive. The reason is that entrepreneurial passion, as a strong emotional expression formed from the inside out by the core team of entrepreneurs (Stenholm and Nielsen, 2019), can stimulate the willpower, innovation, and execution of the entire team in the entrepreneurial process (Obschonka et al., 2019). So entrepreneurial passion can make the entrepreneurial bricolage actions of new ventures more challenging and feasible and accelerate the creation of entrepreneurial opportunities driven by high-speed resource orchestration.

### Practical implications

(1) This study explores the cognitive causes behind entrepreneurial opportunity recognition, specifying the importance of the scanning and search, association and connection, evaluation, and judgment dimensions of entrepreneurial vigilance in opportunity recognition. First, scanning and searching for information expands entrepreneurs' information perception. In practice, entrepreneurs need to develop their sensitivity and alertness to information and enhance their ability to search for knowledge, information, and resources. Second, the association and connection of information deepen the entrepreneur's cognition of new information and expand the possibility of information association. In the process of entrepreneurship, entrepreneurs should cultivate their creativity, broaden their cognitive horizons, and pay attention to the details related to multiple pieces of information with an appropriate degree of distraction. In this way, entrepreneurs can control the information as a whole, connect a piece of information to the whole picture and create innovative connections. Thirdly, evaluation and judgment allow for selecting valid information and assessing potential opportunities in the information. Since the evaluator's subjective knowledge prototype and cognitive logic can influence the outcome of evaluation and judgment, entrepreneurs should think and evaluate information rationally. At the same time, they should enhance their cognitive flexibility and adapt their cognitive model to the external environment to improve the accuracy of entrepreneurs' judgment on the value of information and opportunities.

(2) Entrepreneurial bricolage can create and explore entrepreneurial opportunities through reconfiguring and orchestrating internal resources (Yu et al., 2019), which has a complementary effect on external opportunity identification through entrepreneurial alertness. Alert entrepreneurs focus on valuable information in the market environment and remain highly alert to potential entrepreneurial opportunities but tend to neglect the opportunity creation of the entrepreneurial bricolage process through the resources at hand. Celtekligil (2020) pointed out that the perspective of internal opportunity resource integration tends to “bricolage the resources at hand to create new opportunities” and “allocate the resources at hand to find new opportunities”. At this time, opportunities are created through internal resources. In the entrepreneurial process, entrepreneurs need to search for crucial external information to identify new opportunities, pay attention to the creative integration of resources at hand and give full play to their subjective initiative (Davidsson et al., 2017). Companies need to create opportunities by creatively bricolage and reconfiguring new internal elements, creating and discovering more new opportunities through an “internal and external” approach. In terms of the choice of the specific bricolage, this study recommends a planned bricolage. The main reason is that planned bricolage favors focus, concentrating on available resources and investing limited resources in promising projects. Compared to improvised bricolage, using available resources is more focused and easier to develop resource advantages.

(3) As a strong and positive emotion, entrepreneurial passion has a motivational effect on entrepreneurial bricolage activities. In the process of entrepreneurship, on the one hand, entrepreneurs should actively participate in activities such as entrepreneurial training and outreach training and take the initiative to communicate and share entrepreneurial experiences with other entrepreneurs. In this way, they can strengthen their sense of identity and get positive feedback on the emotional contagion effect, which is conducive to stimulating entrepreneurial passion (Santos G. et al., 2020). On the other hand, entrepreneurs should enhance their entrepreneurial self-efficacy by listening to the thriving entrepreneurial experiences of others. At the same time, they should understand the failure phenomenon in the process of entrepreneurship in advance, control their emotions rationally in the face of failure experience, and strengthen their determination to overcome entrepreneurial setbacks and difficulties, which helps to sustain their entrepreneurial passion (Karimi, 2020).

### Research limitations and outlook

First, there was some unevenness in selecting the study sample due to the epidemic situation and resource constraints. Although the study considered the variability caused by the research regions according to the research reports of authoritative institutions, there are still many regions we have not researched. This imbalance may limit the universal application value of the study findings to some extent, so the follow-up study should pay attention to the sample distribution should be more evenly distributed to carry out cross-regional comparative studies. Secondly, the measurement of the main variables in this study is subjective self-evaluation by entrepreneurs, which may lead to partial deviation. Later studies can use more objective methods to measure and further verify the results of this study. Third, the study used planned and improvised bricolage for model validation in this paper's division of the entrepreneurial bricolage dimension. Future research may consider the different effects of other dimensional segmentation methods on the results.

## Data availability statement

The raw data supporting the conclusions of this article will be made available by the authors, without undue reservation.

## Ethics statement

Ethics review and approval/written informed consent was not required as per local legislation and institutional requirements.

## Author contributions

ZL and BJ: conceptualization. ZL and SB: methodology. ZL: software, writing and original draft preparation, investigation, and data curation. BJ: funding acquisition. BJ, SB, and JF: reviewing. SB and JF: guidance. QC: reviewing. All authors have read and agreed to the published version.

## References

[B1] Amato C. Baron R. A. Barbieri B. Belanger J. J. Pierro A. (2017). Regulatory modes and entrepreneurship: the mediational role of alertness in small business success. J. Small Bus. Manag. 55, 27–42. 10.1111/jsbm.12255

[B2] An W. Xu Y. Zhang J. (2018). Resource constraints, innovation capability and corporate financial fraud in entrepreneurial firms. Chinese Manag. Stud. 12, 2–18. 10.1108/CMS-02-2017-0024

[B3] Anjum T. Heidler P. Amoozegar A. Anees R. T. (2021). The impact of entrepreneurial passion on the entrepreneurial intention; moderating impact of perception of university support. Adm. Sci. 11, 45. 10.3390/admsci11020045

[B4] Ardichvili A. Cardozo R. Ray S. (2003). A theory of entrepreneurial opportunity identification and development. J. Bus. Ventur. 18, 105–123. 10.1016/S0883-9026(01)00068-431244736

[B5] Bacq S. Lumpkin G. T. (2021). Social entrepreneurship and COVID-19. J. Manag. Stud. 58, 285–288. 10.1111/joms.12641

[B6] Bacq S. Ofstein L. F. Kickul J. R. Gundry L. K. (2017). Perceived entrepreneurial munificence and entrepreneurial intentions: a social cognitive perspective. *Int. Small Bus*. J 35, 639–659. 10.1177/0266242616658943

[B7] Baker T. (2007). Resources in play: bricolage in the Toy Store(y). J. Bus Ventur. 22, 694–7111. 10.1016/j.jbusvent.2006.10.008

[B8] Baker T. Miner A. S. Eesley D. T. (2003). Improvising firms: bricolage, account giving and improvisational competencies in the founding process. Res. Policy. 32, 255–276. 10.1016/S0048-7333(02)00099-9

[B9] Baker T. Nelson R. E. (2005). Creating something from nothing: resource construction through entrepreneurial bricolage. Adm. Sci. Q. 50, 329–366. 10.2189/asqu.2005.50.3.329

[B10] Baron R. A. Ensley M. D. (2006). Opportunity recognition as the detection of meaningful patterns: evidence from comparisons of novice and experienced entrepreneurs. Manage. Sci. 52, 1331–1344. 10.1287/mnsc.1060.0538

[B11] Barrett L. F. (2013). Psychological construction: the darwinian approach to the science of emotion. Emotion Rev. 5, 379–389. 10.1177/1754073913489753

[B12] Bennett N. Lemoine G. J. (2014). What VUCA really means for you. Harv. Bus. Rev. 92, 2014.

[B13] Bignetti B. Santos A. C. M. Z. Hansen P. B. Henriqson E. (2021). The influence of entrepreneurial passion and creativity on entrepreneurial intentions. RAM. Revista de Administração Mackenzie. 22, 1–32. 10.1590/1678-6971/eramr210082

[B14] Biraglia A. Kadile V. (2017). The role of entrepreneurial passion and creativity in developing entrepreneurial intentions: insights from American Homebrewers. J. Small bus. Manage. 55, 170–188. 10.1111/jsbm.12242

[B15] Boone S. Andries P. Clarysse B. (2020). Does team entrepreneurial passion matter for relationship conflict and team performance? On the importance of fit between passion focus and venture development stage. J. Bus. Ventur. 35, 105984. 10.1016/j.jbusvent.2019.105984

[B16] Brown R. Mawson S. Rowe A. (2019). Start-ups, entrepreneurial networks and equity crowdfunding: a processual perspective. Ind. Mark. Manag. 80, 115–125. 10.1016/j.indmarman.2018.02.003

[B17] Busenitz L. W. Plummer L. A. Klotz A. C. Shahzad A. Rhoads K. (2014). Entrepreneurship research (1985–2009) and the emergence of opportunities. Entrep. Theory Pract. 38, 1–20. 10.1111/etap.12120

[B18] Campos H. M. (2021). Strategic orientations, hypercompetitive environment, and entrepreneurial alertness of small firms: evidence from the central region of Mexico. Int. J. Entrepreneurship Small Bus. 42, 532. 10.1504/IJESB.2021.114242

[B19] Cardon M. S. (2008). Is passion contagious? The transference of entrepreneurial passion to employees. Hum. Resour. Manag. Rev. 18, 77–86. 10.1016/j.hrmr.2008.04.001

[B20] Cardon M. S. Kirk C. P. (2015). Entrepreneurial Passion as Mediator of the Self–Efficacy to Persistence Relationship. Entrep. Theory Pract. 39, 1027–1050. 10.1111/etap.12089

[B21] Cardon M. S. Post C. Forster W. R. (2017). Team entrepreneurial passion: its emergence and influence in new venture teams. Acad. Manage Rev. 42, 283–305. 10.5465/amr.2014.0356

[B22] Cardon M. S. Sudek R. Mitteness C. (2009a). Frontiers of entrepreneurship research the impact of perceived entrepreneurial passion on angel investing. Front. Entrep. Res. 29, 1.

[B23] Cardon M. S. Wincent J. Singh J. Drnovsek M. (2009b). The nature and experience of entrepreneurial passion. Acad. Manage Rev. 34, 511–532. 10.5465/amr.2009.40633190

[B24] Cavaliere V. Sassetti S. Lombardi S. (2022). Entrepreneurial alertness and self-perceived employability: a virtuous marriage for career development. Personnel Rev. 51, 137–158. 10.1108/PR-05-2020-0350

[B25] Celtekligil K. (2020). “Resource dependence theory,” in Strategic Outlook for Innovative Work Behaviours (Cham: Springer), 131–148.

[B26] Chavoushi Z. H. Zali M. R. Valliere D. Faghih N. Hejazi R. Dehkordi A. M. (2021). Entrepreneurial alertness: a systematic literature review. J. Small Bus. Entrepreneurship. 33, 123–152. 10.1080/08276331.2020.1764736

[B27] Collewaert V. Anseel F. Crommelinck M. de Beuckelaer A. Vermeire J. (2016). When passion fades: disentangling the temporal dynamics of entrepreneurial passion for founding. J. Manag. Stud. 53, 966–995. 10.1111/joms.12193

[B28] Companys Y. E. McMullen J. S. (2007). Strategic entrepreneurs at work: the nature, discovery, and exploitation of entrepreneurial opportunities. Small Bus. Econ. 28, 301–322. 10.1007/s11187-006-9034-x

[B29] Cosenz F. Noto G. (2018). Fostering entrepreneurial learning processes through Dynamic Start-up business model simulators. Int. J. Educ. Manag. 16, 468–482. 10.1016/j.ijme.2018.08.003

[B30] Crespo N. F. Simões V. C. Fontes M. (2022). Uncovering the factors behind new ventures' international performance: capabilities, alertness and technological turbulence. Eur. Manag. J. 40, 344–359. 10.1016/j.emj.2021.07.009

[B31] Daniel A. D. Adeel S. Botelho A. (2021). Entrepreneurial alertness research: past and future. Sage Open. 1121582440211031535. 10.1177/21582440211031535

[B32] Davidsson P. Baker T. Senyard J. M. (2017). A measure of entrepreneurial bricolage behavior. Int. J. Entrepreneurial Behav. Res. 23, 114–135. 10.1108/IJEBR-11-2015-025627259179

[B33] de Carolis D. M. Saparito P. (2006). Social capital, cognition, and entrepreneurial opportunities: a theoretical framework. Entrep. Theory Pract. 30, 41–56. 10.1111/j.1540-6520.2006.00109.x

[B34] de Mol E. Cardon M. S. de Jong B. Khapova S. N. Elfring T. (2020). Entrepreneurial passion diversity in new venture teams: an empirical examination of short- and long-term performance implications. J. Bus. Ventur. 35, 105965. 10.1016/j.jbusvent.2019.105965

[B35] Dheer R. J. S. Lenartowicz T. (2019). Cognitive flexibility: impact on entrepreneurial intentions. J. Vocat. Behav. 115, 103339. 10.1016/j.jvb.2019.103339

[B36] Duymedjian R. Rüling C-C. (2010). Towards a foundation of bricolage in organization and management theory. Organ. Stud. 31, 133–151. 10.1177/0170840609347051

[B37] Ekelund R. B. Kirzner I. M. (1974). Competition and entrepreneurship. South. Econ. J. 41, 155. 10.2307/1056112

[B38] El-Awad Z. Gabrielsson J. Politis D. (2017). Entrepreneurial learning and innovation. Int. J. Entrepreneurial Behav. Res. 23, 381–405. 10.1108/IJEBR-06-2016-0177

[B39] Fatoki O. (2014). The Entrepreneurial Alertness of Immigrant Entrepreneurs in South Africa. Mediterr. J. Soc. Sci. 5, 722–722. 10.5901/mjss.2014.v5n23p722

[B40] Feng B. Chen M. (2020). The impact of entrepreneurial passion on psychology and behavior of entrepreneurs. Front. Psychol. 11,1733. 10.3389/fpsyg.2020.01733 32793066 PMC7385187

[B41] Fisher G. (2012). Effectuation, causation, and bricolage: a behavioral comparison of emerging theories in entrepreneurship research. Entrep. Theory Pract. 36, 1019–1051. 10.1111/j.1540-6520.2012.00537.x

[B42] Foss N. J. Klein P. G. (2010). Alertness, action, and the antecedents of entrepreneurship. J. Priv. Enterp. 25, 145. 10.2139/ssrn.1579975

[B43] Gaglio C. M. Katz J. A. (2001). The psychological basis of opportunity identification: entrepreneurial alertness. Small Bus. Econ. 16, 95–111. 10.1023/A:1011132102464

[B44] Gancarczyk M. Ujwary-Gil A. (2021). Entrepreneurial cognition or judgment: the management and economics approaches to the entrepreneur's choices. J. Entrepreneurship Innov. 17, 7–23. 10.7341/20211710

[B45] Gill S. A. Bencheva N. Karayel S. Usman M. (2021). Does entrepreneurial self-efficacy moderate effects of cognitive flexibility and entrepreneurial alertness on entrepreneurial intentions? Entrepreneurial Bus. Econ. Rev. 9, 25–41. 10.15678/EBER.2021.090302

[B46] Guo H. Su Z. Ahlstrom D. (2016). Business model innovation: the effects of exploratory orientation, opportunity recognition, and entrepreneurial bricolage in an emerging economy. Asia Pac. J. Manag. 33, 533–549. 10.1007/s10490-015-9428-x

[B47] He C. Lu J. Qian H. (2019). Entrepreneurship in China. Small Bus. Econ. 52, 563–572. 10.1007/s11187-017-9972-5

[B48] Hooi H. C. Ahmad N. H. Amran A. Rahman S. A. (2016). The functional role of entrepreneurial orientation and entrepreneurial bricolage in ensuring sustainable entrepreneurship. Manag. Res. Rev. 39, 1616–1638. 10.1108/MRR-06-2015-0144

[B49] Hou F. Qi M.-D. Su Y. Tan X.-X. Yang B.-X. (2022). Trickle-down effects of entrepreneurial bricolage and business model innovation on employee creativity: evidence from entrepreneurial internet firms in China. Front. Psychol. 12,801202. 10.3389/fpsyg.2021.801202 35185699 PMC8847182

[B50] Hou F. Su Y. Lu M. Qi M. (2019). Model of the entrepreneurial intention of university students in the Pearl River Delta of China. Front. Psychol. 10. 10.3389/fpsyg.2019.00916 31114522 PMC6503157

[B51] Huy Q. Zott C. (2019). Exploring the affective underpinnings of dynamic managerial capabilities: How managers' emotion regulation behaviors mobilize resources for their firms. Strateg. Manag. J. 40, 28–54. 10.1002/smj.2971

[B52] Ireland R. (2003). A model of strategic entrepreneurship: the construct and its dimensions. J. Manage. 29, 963–989. 10.1016/S0149-2063(03)00086-2

[B53] Kadile V. Biraglia A. (2022). From hobby to business: exploring environmental antecedents of entrepreneurial alertness using fsQCA. J. Small Bus. Manage. 60, 580–615. 10.1080/00472778.2020.1719846

[B54] Karimi S. (2020). The role of entrepreneurial passion in the formation of students' entrepreneurial intentions. Appl. Econ. 52, 331–344. 10.1080/00036846.2019.1645287

[B55] Kirtley J. O'Mahony S. (2020). What is a pivot? Explaining when and how entrepreneurial firms decide to make strategic change and pivot. Strateg. Manag. J. 1–34. 10.1002/smj.3131

[B56] Kirzner I. M. (2009). The alert and creative entrepreneur: a clarification. Small Bus. Econ. 32, 145–152. 10.1007/s11187-008-9153-7

[B57] Korsgaard M. A. Roberson L. (1995). Procedural justice in performance evaluation: the role of instrumental and non-instrumental voice in performance appraisal discussions. J. Manage. 21, 657–669. 10.1177/014920639502100404

[B58] Lanivich S. E. Smith A. Levasseur L. Pidduck R. J. Busenitz L. Tang J. (2022). Advancing entrepreneurial alertness: review, synthesis, and future research directions. J. Bus. Res. 139, 1165–1176. 10.1016/j.jbusres.2021.10.023

[B59] Lee K. Kim Y. Koh D. (2016). Organizational learning, top management team's entrepreneurial alertness, and corporate entrepreneurship in high-tech firms. Asian J. Technol. Innov. 24, 338–360. 10.1080/19761597.2016.1249381

[B60] Lee Y. Herrmann P. (2021). Entrepreneurial passion: a systematic review and research opportunities. J. Bus. Strategy. 31,122–147. 10.53703/001c.29740

[B61] Li C. Murad M. Ashraf S. F. Syed N. Riaz M. (2020a). Entrepreneurial nascent behaviour: the role of causation process in opportunity discovery and creation. Entrepreneurial Bus. Econ. Rev. 8, 27–49. 10.15678/EBER.2020.080410

[B62] Li C. Murad M. Shahzad F. Khan M. A. S. Ashraf S. F. Dogbe C. S. K. (2020b). Entrepreneurial passion to entrepreneurial behavior: role of entrepreneurial alertness, entrepreneurial self-efficacy and proactive personality. Front. Psychol. 11, 1611. 10.3389/fpsyg.2020.01611 32973593 PMC7468520

[B63] Li H. Atuahene-Gima K. (2001). Product innovation strategy and the performance of new technology ventures in China. Acad. Manage J. 44, 1123–1134. 10.2307/3069392

[B64] Li Z. (2013). Entrepreneurial Alertness. Berlin, Heidelberg: Springer Berlin Heidelberg.

[B65] Lin S. Yamakawa Y. Li J. (2019). Emergent learning and change in strategy: empirical study of Chinese serial entrepreneurs with failure experience. Int. Entrepreneurship Manag. J. 15, 773–792. 10.1007/s11365-018-0554-z

[B66] Lumpkin G. T. Lichtenstein B. B. (2005). The role of organizational learning in the opportunity–recognition process. Entrep. Theory Pract. 29, 451–472. 10.1111/j.1540-6520.2005.00093.x

[B67] Luu N. Nguyen H. (2021). Entrepreneurial passion and a firm's innovation strategies. J. Small Bus. Manage. 59, 794–818. 10.1080/00472778.2020.1729026

[B68] Mair J. Marti I. (2007). Entrepreneurship for social impact: encouraging market access in rural Bangladesh. Corp. Gov. 7, 493–501. 10.1108/14720700710820579

[B69] Maran T. K. Bachmann A. K. Mohr C. Ravet-Brown T. Vogelauer L. Furtner M. (2021). Motivational foundations of identifying and exploiting entrepreneurial opportunities. Int. J. Entrepreneurial Behav. Res. 27, 1054–1081. 10.1108/IJEBR-05-2020-0291

[B70] Masango S. G. Lassalle P. (2020). What entrepreneurs do? Entrepreneurial action guided by entrepreneurial opportunities and entrepreneurial learning in early internationalising firms. Int. Mark. Rev. 37, 1083–1119. 10.1108/IMR-10-2018-0273

[B71] Mitchell R. K. Busenitz L. W. Bird B. Marie Gaglio C. McMullen J. S. Morse E. A.. (2007). The central question in entrepreneurial cognition research 2007. Entrep. Theory Pract. 31, 1–27. 10.1111/j.1540-6520.2007.00161.x

[B72] Mole K. F. Adomako S. Tang J. Yu A. (2019). Entrepreneurial orientation and firm performance: the enabling effect of entrepreneurial alertness. Acad. Manag. Ann. 2019, 11480. 10.5465/AMBPP.2019.11480abstract

[B73] Montiel-Campos H. (2021). Moderating role of entrepreneurial alertness on the relationship between entrepreneurial passion and strategic change. J. Organ. Chang. Manag. 34, 1107–1124. 10.1108/JOCM-12-2020-0386

[B74] Morris M. Liguori E. (2016). Annals of Entrepreneurship Education and Pedagogy – 2016. Cheltenham, United Kingdom: Edward Elgar Publishing. 10.4337/9781784719166

[B75] Mueller B. A. Shepherd D. A. (2016). Making the most of failure experiences: exploring the relationship between business failure and the identification of business opportunities. Entrep. Theory Pract. 40, 457–487. 10.1111/etap.12116

[B76] Narayanan V. K. Zane L. J. Liguori E. (2021). Critical methodological considerations for entrepreneurial cognition research. J. Small Bus. Manage. 59, 756–793. 10.1080/00472778.2020.1799634

[B77] Neneh B. N. (2019). From entrepreneurial alertness to entrepreneurial behavior: the role of trait competitiveness and proactive personality. Pers. Individ. Dif. 138, 273–279. 10.1016/j.paid.2018.10.020

[B78] Neneh B. N. (2022). Entrepreneurial passion and entrepreneurial intention: the role of social support and entrepreneurial self-efficacy. High. Educ. Stud. 47, 587–603. 10.1080/03075079.2020.1770716

[B79] Nor-Aishah H. Ahmad N. H. Thurasamy R. (2020). Entrepreneurial leadership and sustainable performance of manufacturing smes in malaysia: the contingent role of entrepreneurial bricolage. Sustainability. 12, 3100. 10.3390/su12083100

[B80] Obschonka M. Hakkarainen K. Lonka K. Salmela-Aro K. (2017). Entrepreneurship as a twenty-first century skill: entrepreneurial alertness and intention in the transition to adulthood. Small Bus. Econ. 48, 487–501. 10.1007/s11187-016-9798-6

[B81] Obschonka M. Moeller J. Goethner M. (2019). Entrepreneurial passion and personality: the case of academic entrepreneurship. Front. Psychol. 9, 2697. 10.3389/fpsyg.2018.02697 30687165 PMC6335975

[B82] Olugbola S. A. (2017). Exploring entrepreneurial readiness of youth and startup success components: entrepreneurship training as a moderator. J. Innov. Knowl. 2, 155–171. 10.1016/j.jik.2016.12.004

[B83] Onwuegbuzie H. N. Mafimisebi O. P. (2021). Global relevance of scaling African indigenous entrepreneurship. Technol. Forecast. Soc. Change 166, 120629. 10.1016/j.techfore.2021.120629

[B84] Phillips N. Tracey P. (2007). Opportunity recognition, entrepreneurial capabilities and bricolage: connecting institutional theory and entrepreneurship in strategic organization. Strateg Organ. 5, 313–320. 10.1177/1476127007079956

[B85] Pidduck R. J. Busenitz L. W. Zhang Y. Ghosh Moulick A. (2020). Oh, the places you'll go: a schema theory perspective on cross-cultural experience and entrepreneurship. J. Bus. Ventur. Insights. 14, e00189. 10.1016/j.jbvi.2020.e00189

[B86] Pirhadi H. Soleimanof S. Feyzbakhsh A. (2021). Unpacking entrepreneurial alertness: how character matters for entrepreneurial thinking. J. Small Bus. Manage., 1–32. 10.1080/00472778.2021.1907584

[B87] Podsakoff P. M. MacKenzie S. B. Lee J.-Y. Podsakoff N. P. (2003). Common method biases in behavioral research: a critical review of the literature and recommended remedies. J. Appl. Psychol. 88, 879–903. 10.1037/0021-9010.88.5.879 14516251

[B88] Rahman S. A. Taghizadeh S. K. Alam M. M. D. Khan G. M. (2020). The functionality of entrepreneurial passion and entrepreneurial bricolage on micro-entrepreneur's wellbeing. J. Small Bus. Strategy. 30, 47–64.

[B89] Ramoglou S. (2021). Why do disequilibria exist? An ontological study of Kirznerian economics. Cambridge J. Econ. 45, 833–856. 10.1093/cje/beab015

[B90] Renko M. Shrader R. C. Simon M. (2012). Perception of entrepreneurial opportunity: a general framework. Manage. Deci. 50, 1233–1251. 10.1108/00251741211246987

[B91] Rippa P. Secundo G. (2019). Digital academic entrepreneurship: the potential of digital technologies on academic entrepreneurship. Technol. Forecast. Soc. Change 146, 900–911. 10.1016/j.techfore.2018.07.01320630383

[B92] Rocha V. Carneiro A. Amorim Varum C. (2015). Serial entrepreneurship, learning by doing and self-selection. Int J Ind Organ 40, 91–106. 10.1016/j.ijindorg.2015.04.001

[B93] Roy N. (2020). Uncertainty as Entrepreneurial Motivation: Tuche, karma and the Necessity of Action. Philos Manag. 19, 89–98. 10.1007/s40926-019-00122-z

[B94] Sahut J.-M. Iandoli L. Teulon F. (2021). The age of digital entrepreneurship. Small Bus. Econ. 56, 1159–1169. 10.1007/s11187-019-00260-8

[B95] Salunke S. Weerawardena J. McColl-Kennedy J. R. (2013). Competing through service innovation: the role of bricolage and entrepreneurship in project-oriented firms. J. Bus. Res. 66, 1085–1097. 10.1016/j.jbusres.2012.03.005

[B96] Santos G. Marques C. S. Ferreira J. J. M. (2020). Passion and perseverance as two new dimensions of an Individual Entrepreneurial Orientation scale. J. Bus. Res. 112, 190–199. 10.1016/j.jbusres.2020.03.016

[B97] Santos S. C. Caetano A. Costa S. F. Rueff Lopes R. Silva A. J. Neumeyer X. (2020). Uncovering the affective turmoil during opportunity recognition and exploitation: a nonlinear approach. J. Bus. Ventur. Insights. 14, e00184. 10.1016/j.jbvi.2020.e00184

[B98] Santos S. C. Cardon M. S. (2019). What's love got to do with it? Team entrepreneurial passion and performance in new venture teams. Entrep. Theory Pract. 43, 475–504. 10.1177/1042258718812185

[B99] Sassetti S. Cavaliere V. Lombardi S. (2022). The rhythm of effective entrepreneurs' decision-making process. The pathways of alertness scanning and search and cognitive style. A mediation model. Int. Entrepreneurship Manag. J. 18, 555–578. 10.1007/s11365-021-00759-1

[B100] Sassetti S. Marzi G. Cavaliere V. Ciappei C. (2018). Entrepreneurial cognition and socially situated approach: a systematic and bibliometric analysis. Scientometrics. 116, 1675–1718. 10.1007/s11192-018-2809-4

[B101] Senyard J. Baker T. Davidsson P. (2009). Entrepreneurial bricolage: towards systematic empirical testing. Front. Entrep. Res. 29, 5.

[B102] Shabbir S. Danish R. Q. Rehman M. Hasnain M. Asad H. (2021). An empirical investigation of environmental turbulence and fear in predicting entrepreneurial improvisation. J. Open Innov.: Technol. Mar. 7, 157. 10.3390/joitmc7020157

[B103] Shaheen I. Azadegan A. Davis D. F. (2022). Resource scarcity and humanitarian social innovation: observations from hunger relief in the context of the COVID-19 pandemic. J. Bus. Ethics. 1–21. 10.1007/s10551-021-05014-9 35035004 PMC8747850

[B104] Shane S. A. (2003). A General Theory of Entrepreneurship: The Individual-Opportunity Nexus. Edward Elgar Publishing.

[B105] Sharma L. (2019). A systematic review of the concept of entrepreneurial alertness. J. Entrepreneurship Emerg. Econ. 11, 217–233. 10.1108/JEEE-05-2018-0049

[B106] Shepherd D. A. Mcmullen J. S. Ocasio W. (2017). Is that an opportunity? An attention model of top managers' opportunity beliefs for strategic action. Strateg. Manag. J. 38, 626–644. 10.1002/smj.2499

[B107] Sirmon D. G. Hitt M. A. Ireland R. D. Gilbert B. A. (2011). Resource orchestration to create competitive advantage. J. Manage. 37, 1390–1412. 10.1177/014920631038569533670749

[B108] Smith J. B. Mitchell J. R. Mitchell R. K. (2009). Entrepreneurial scripts and the new transaction commitment mindset: extending the expert information processing theory approach to entrepreneurial cognition research. Entrep. Theory Pract. 33, 815–844. 10.1111/j.1540-6520.2009.00328.x

[B109] Steffens P. R. Baker T. Davidsson P. Senyard J. M. (2022). When Is less more? Boundary conditions of effective entrepreneurial bricolage. J Manage. 01492063221077210. 10.1177/01492063221077210

[B110] Stenholm P. Nielsen M. S. (2019). Understanding the emergence of entrepreneurial passion. Int. J. Entrepreneurial Behav. Res. 25, 1368–1388. 10.1108/IJEBR-02-2018-0065

[B111] Stevenson H. H. Jarrillo-Mossi J. C. (1986). Preserving entrepreneurship as companies grow. J. Bus. Strategy. 7, 10–23. 10.1108/eb039138

[B112] Sunduramurthy C. Zheng C. Musteen M. Francis J. Rhyne L. (2016). Doing more with less, systematically? Bricolage and ingenieuring in successful social ventures. J. World Bus. 51, 855–870. 10.1016/j.jwb.2016.06.005

[B113] Syed I. Butler J. C. Smith R. M. Cao X. (2020). From entrepreneurial passion to entrepreneurial intentions: The role of entrepreneurial passion, innovativeness, and curiosity in driving entrepreneurial intentions. Pers. Individ. Dif. 157, 109758. 10.1016/j.paid.2019.109758

[B114] Tang J. (2016). Linking personal turbulence and creative behavior: the influence of scanning and search in the entrepreneurial process. J. Bus. Res. 69, 1167–1174. 10.1016/j.jbusres.2015.09.017

[B115] Tang J. Kacmar K. M. Busenitz L. (2012). Entrepreneurial alertness in the pursuit of new opportunities. J. Bus. Ventur. 27, 77–94. 10.1016/j.jbusvent.2010.07.001

[B116] Timmons J. A. Spinelli S. Tan Y. (2004). New Venture Creation: Entrepreneurship for the 21st Century, Vol. 6. New York, NY: McGraw-Hill/Irwin.

[B117] Tindiwensi C. K. Abaho E. Munene J. C. Muhwezi M. Nkote I. N. (2021). Entrepreneurial bricolage in smallholder commercial farming: a family business perspective. J. Fam. Bus. Strategy. 11, 423–439. 10.1108/JFBM-04-2020-0036

[B118] Ucbasaran D. Westhead P. Wright M. (2009). The extent and nature of opportunity identification by experienced entrepreneurs. J. Bus. Ventur. 24, 99–115. 10.1016/j.jbusvent.2008.01.008

[B119] Urban B. Wood E. (2017). The innovating firm as corporate entrepreneurship. Eur. J. Innov. Manag. 20, 534–556. 10.1108/EJIM-10-2016-010034168600

[B120] Uygur U. (2019). An analogy explanation for the evaluation of entrepreneurial opportunities. J. Small Bus. Manage. 57, 757–779. 10.1111/jsbm.12321

[B121] Vallerand R. J. Mageau G. A. Ratelle C. Léonard M. Blanchard C. Koestner R.. (2003). Les Passions de 1'Âme: on obsessive and harmonious passion. J. Pers. Soc. Psychol. 85, 756. 10.1037/0022-3514.85.4.756 14561128

[B122] Valliere D. (2013). Towards a schematic theory of entrepreneurial alertness. J. Bus. Ventur. 28, 430–442. 10.1016/j.jbusvent.2011.08.004

[B123] van de Sandt N. Mauer R. (2019). The effects of action-based entrepreneurship education on ambiguity tolerance and entrepreneurial alertness. J. Entrep. Educ. 22, 1–12.

[B124] Wang X. Yu X. Meng X. (2021). Entrepreneurial bricolage and new product development performance in new ventures: the contingent effects of founding team involvement. Entrepreneurship Res. J. 20200485. 10.1515/erj-2020-0485

[B125] Welter C. Mauer R. Wuebker R. J. (2016). Bridging behavioral models and theoretical concepts: effectuation and bricolage in the opportunity creation framework. Strateg. Entrep. J. 10, 5–20. 10.1002/sej.1215

[B126] Witell L. Gebauer H. Jaakkola E. Hammedi W. Patricio L. Perks H. (2017). A bricolage perspective on service innovation. J. Bus. Res. 79, 290–298. 10.1016/j.jbusres.2017.03.021

[B127] Yang M. (2018). International entrepreneurial marketing strategies of MNCs: bricolage as practiced by marketing managers. Int. Bus. Rev. 27, 1045–1056. 10.1016/j.ibusrev.2018.03.004

[B128] Yu X. Li Y. Chen D. Q. Meng X. Tao X. (2019). Entrepreneurial bricolage and online store performance in emerging economies. Electronic Mark. 29, 167–185. 10.1007/s12525-018-0302-9

[B129] Yu X. Li Y. Su Z. Tao Y. Nguyen B. Xia F. (2020). Entrepreneurial bricolage and its effects on new venture growth and adaptiveness in an emerging economy. Asia Pac. J. Manag. 37, 1141–1163. 10.1007/s10490-019-09657-1

[B130] Zaichkowsky J. L. (1985). Measuring the involvement construct. J. Consum. Res. 12, 341. 10.1086/208520

